# Phylogeny of Transferable Oxazolidinone Resistance Genes and Homologs

**DOI:** 10.3390/antibiotics13040311

**Published:** 2024-03-28

**Authors:** Gábor Kardos, Levente Laczkó, Eszter Kaszab, Bálint Timmer, Krisztina Szarka, Eszter Prépost, Krisztián Bányai

**Affiliations:** 1 Institute of Metagenomics, University of Debrecen, H-4032 Debrecen, Hungary; timmer.balint@pte.hu (B.T.); szkrisz@med.unideb.hu (K.S.); 2 One Health Institute, Faculty of Health Sciences, University of Debrecen, H-4032 Debrecen, Hungary; laczko.levente@med.unideb.hu (L.L.); kaszab.eszter@univet.hu (E.K.); 3 HUN-REN-DE Conservation Biology Research Group, H-4032 Debrecen, Hungary; 4 Department of Microbiology and Infectious Diseases, University of Veterinary Medicine, H-1078 Budapest, Hungary; 5 Department of Medical Microbiology and Immunology, University of Pécs, H-7624 Pécs, Hungary; 6 Department of Health Industry, University of Debrecen, H-4032 Debrecen, Hungary; prepost.eszter@unideb.hu; 7 Pathogen Discovery Group, HUN-REN Veterinary Medical Research Institute, H-1143 Budapest, Hungary; 8 National Laboratory for Infectious Animal Diseases, Antimicrobial Resistance, Veterinary Public Health and Food Chain Safety, H-1143 Budapest, Hungary; 9 Department of Pharmacology and Toxicology, University of Veterinary Medicine, H-1078 Budapest, Hungary

**Keywords:** cfr methylase, ribosome methylation, 23S rRNA, linezolid resistance, phenicol resistance

## Abstract

Oxazolidinone resistance, especially transmissible resistance, is a major public health concern, and the origin of this resistance mechanism is not yet resolved. This study aims to delve into the phylogenetic origin of the transmissible oxazolidinone resistance mechanisms conferring cross-resistance to other drugs of human and veterinary importance. The amino acid sequences of the five cfr ribosomal methylases and optrA and poxtA were used as queries in searches against 219,549 bacterial proteomes in the NCBI RefSeq database. Hits with >40% amino acid identity and >80% query coverage were aligned, and phylogenetic trees were reconstructed. All five cfr genes yielded highly similar trees, with rlmN housekeeping ribosomal methylases located basal to the sister groups of S-adenosyl-methionine-dependent methyltransferases from various Deltaproteobacteria and Actinomycetia, including antibiotic-producing *Streptomyces* species, and the monophyletic group of cfr genes. The basal branches of the latter contained paenibacilli and other soil bacteria; they then could be split into the clades [cfr(C):cfr(E)] and [[cfr:cfr(B)]:cfr(D)], always with different Bacillaceae in their stems. Lachnospiraceae were encountered in the basal branches of both optrA and poxtA trees. The ultimate origin of the cfr genes is the rlmN housekeeping ribosomal methylases, which evolved into a suicide-avoiding methylase in antibiotic producers; a soil organism (Lachnospiraceae, Paenibacilli) probably acted as a transfer organism into pathogenic bacteria. In the case of optrA, the porcine pathogenic *Streptococcus suis* was present in all branches, while the proteins closest to poxtA originated from Clostridia.

## 1. Introduction

The oxazolidinones linezolid and tedizolid are the last-resort drugs against multi-resistant Gram-positive bacteria, i.e., methicillin-resistant staphylococci and vancomycin-resistant enterococci (VRE). Therefore, oxazolidinone resistance is a great public health concern, especially transmissible resistance, which can spread rapidly among commensals and pathogens. Oxazolidinone resistance, which is due to a target mutation (i.e., a G2576T substitution in domain V of 23S rRNA), emerged within a year of linezolid commercialization [1]. Since then, several other mutations have been reported, some of which have led to cross-resistance to other drug groups that target the large subunit of the ribosome, such as phenicols [2]. In this manner, these drugs can induce resistance to oxazolidinones [3]. As phenicols are extensively used in veterinary medicine, this case exemplifies how animals can serve as evolutionary hotspots for the development of resistance to antibiotics used exclusively in humans [3,4]. Although the problem of the zoonotic spread of resistant mutants is alleviated by the difficulty of host-switching in many bacterial species of concern [5,6], transmissible resistance genes that reside on and spread through mobile genetic elements pose an even greater problem. For example, the appearance of *cfr* genes in clinical *Staphylococcus* isolates encoding an RNA methylase that confers resistance to phenicols, lincosamides, Streptogramin A, pleuromutilins and oxazolidinones has raised serious concerns [1,7,8]. As many of these drugs (with the exception of oxazolidinones) are widely used in veterinary medicine, including aquaculture [9,10], it has been hypothesized that this gene has arisen and spread in potential pathogens due to the extensive use of these compounds [3,11,12]. Since the first report of staphylococci/mammaliicocci [13], this gene has been found in enterococci and Streptococci, and other related genes, namely, *cfr*(B), *cfr*(C), *cfr*(D) and *cfr*(E) have also been described in enterococci and in *Clostridioides difficile*, as well as in several other bacteria [14].

In addition to the *cfr* genes, other transmissible genes such as *optrA* and *poxtA* have been described as associated with resistance to both commercialized oxazolidinones [15,16]. These encode ATP-binding cassette transporters, and resistance is conferred by ribosomal protection [17]. It is clear that optrA provide resistance to oxazolidinones and phenicols and was first found in enterococci [15], while poxtA also mediates resistance to phenicols and tetracyclines (including tigecycline), first described in *Staphylococcus aureus* [16]. Their presence in Gram-negative bacteria has also been documented [18]. These genes can also be co-harbored on the same plasmid [19].

Although there are hypotheses about the origin of these resistance mechanisms, the evolutionary origin of these genes is not yet clear. This paper addresses the phylogeny of transmissible linezolid-resistant genes using phylogenetic methods.

## 2. Results

**Figure 1:**
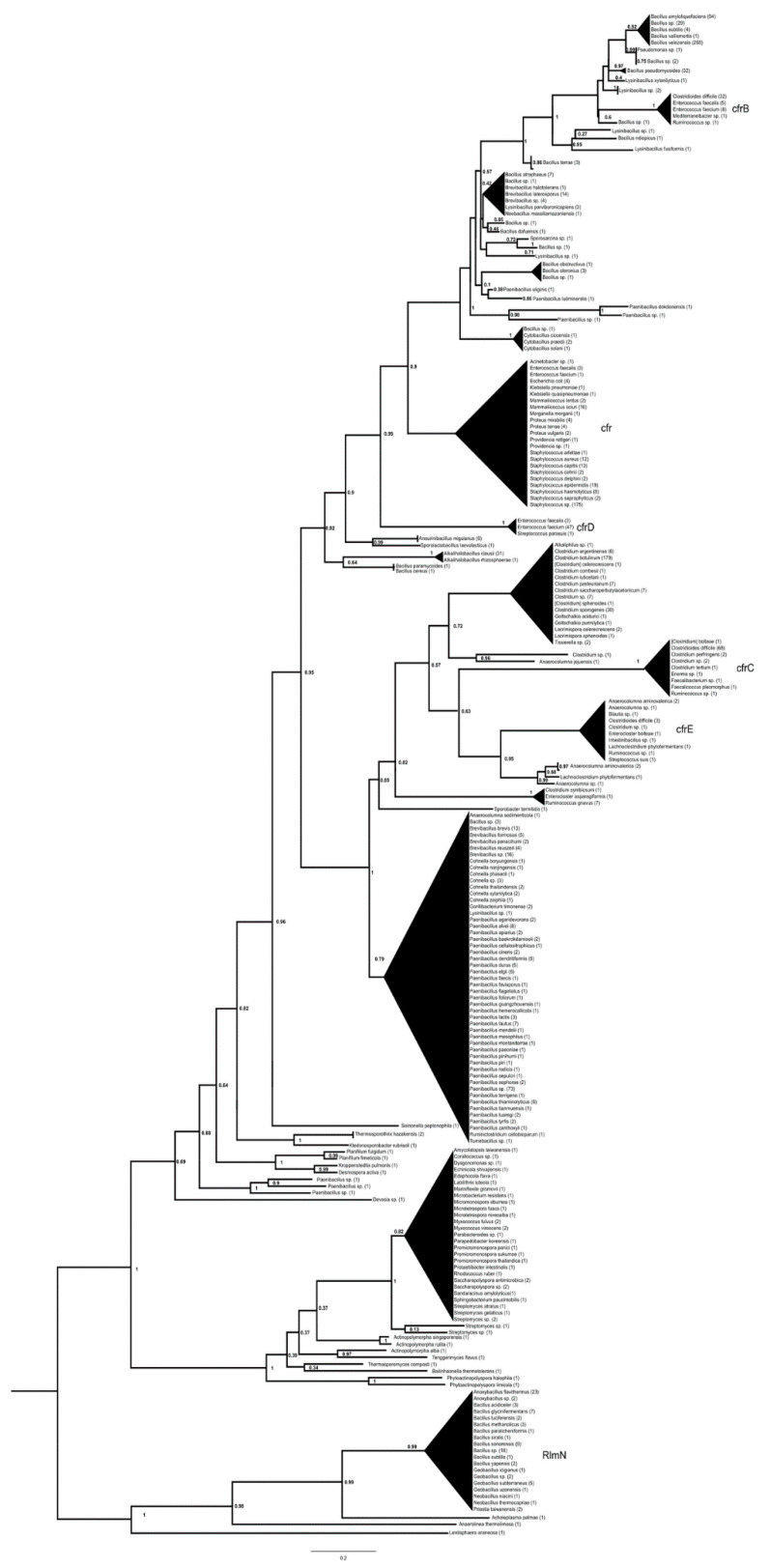
Phylogeny of genes with cfr (NG070225.1) as a query reconstructed with FastTree using the amino acid hits of the reference gene sequence with matches of >40% amino acid identity and >85% query coverage. The numbers above represent SH-like aLRT support values.

The basal split in the cfr phylogeny was found between rlmN housekeeping methylases and their sister group, which consisted of two major clades. One clade contained S-adenosyl-methionine-dependent methyltransferases from various Deltaproteobacteria and Actinomycetia, including the antibiotic-producing *Streptomyces* species; the other major clade comprised cfr proteins. In the latter clade, the most basal branch separated the soil alphaproteobacterium *Devosia* from proteins of *Paenibacillus* sp. and various soil-dwelling members of the Thermoactinomycetaceae and Chloroflexi (Figure 1, Supplementary Materials Figure S1). This clade then split into two large sister groups, with the basal branches in both groups containing proteins from different Bacillales.

One of these contained proteins from *Paenibacillus*, *Brevibacillus* and *Cohnella*, then a branch-containing protein from *Ruminococcus* split off, and a group with poorly resolved branches consisted of proteins from various *Clostridium* spp., including *C. botulinum*, cfrC proteins from *Clostridioides difficile* and a few other species (*Faecalicoccus*, *Ruminococcus*, *Enterocloster*, *C. perfringens*) as well as several cfrE proteins from *C. difficile*, *Streptococcus suis*, *Intestinibacillus* sp., *Blautia* sp. and *Enterocloster bolteae*.

The basal taxa in the other group were *Bacillus cereus*, *B. paramycoides*, *Aneurinibacillus* and *Sporolactobacillus*; the next branch consisted of cfr(D) proteins from an *S. parasuis* and a group of *Enterococcus faecium* isolates. A further branch contained cfr proteins from clinically relevant staphylococci (*S. aureus*, *S. haemolyticus*, *S. epidermidis*, *S. capitis*), *Mammaliicoccus sciuri*, *Enterococcus faecium*, *E. faecalis* and various Enterobacterales (all annotated as cfr). Further branches formed a complex structure of proteins from various Bacillales (*Cytobacillus*, *Paenibacillus*, *Bacillus*, *Lysinibacillus*, *Brevibacillus*), with the clade of cfr(B) containing proteins from *C. difficile*, *Enterococcus faecalis*, *E. faecium*, *Mediterraneibacter* and *Ruminococcus*. The most-derived branch included clbA rRNA methylase proteins related to cfrs from various *Bacillus* spp. including *B. amyloliquefaciens* and *B. velezensis*.

Queries of cfr(B), cfr(C), cfr(D) and cfr(E) yielded very similar trees, with the basal rlmN proteins showing the greatest differences. In the case of cfr(B), these were completely absent, and the root of the tree separated the S-adenosyl-methionine-dependent methyltransferases and cfrs; the cfr(D) query yielded only five rlmN proteins in the base of the tree, some of which differed from those in the cfr tree; cfr(E) yielded a tree that was virtually identical in topology to the tree with cfr as the query.

**Figure 2:**
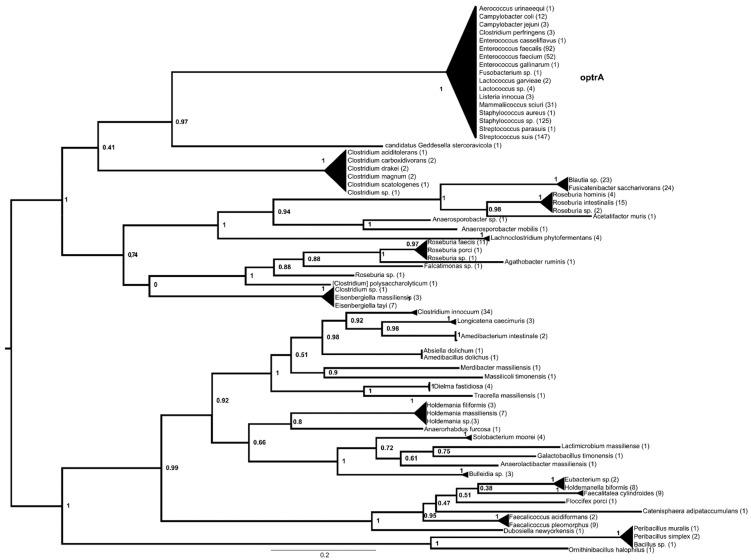
Phylogeny of genes with optrA (MF805732.1:8426-10393) as a query reconstructed with FastTree using the amino acid hits of the reference gene sequence with matches of >40% amino acid identity and >85% query coverage. The numbers above represent SH-like aLRT support values.

The optrA phylogeny contained two major lineages (Figure 2, Supplementary Materials Figure S2), one containing *Peribacillus*-derived proteins, followed by branches containing proteins from various Erysipelotrichaceae associated with the human microbiota. The other major lineage was divided into two sister groups, one containing proteins from various Lachnospiraceae associated with the human and animal microbiota, while the other contained proteins from soil-associated *Clostridium* spp. and proteins from clinically important bacteria, i.e., enterococci, staphylococci, *Mammaliicoccus*, *Lactococcus*, *Listeria*, *Campylobacter* and *Streptococcus suis*, with the latter appearing in most recognizable subgroups.

**Figure 3:**
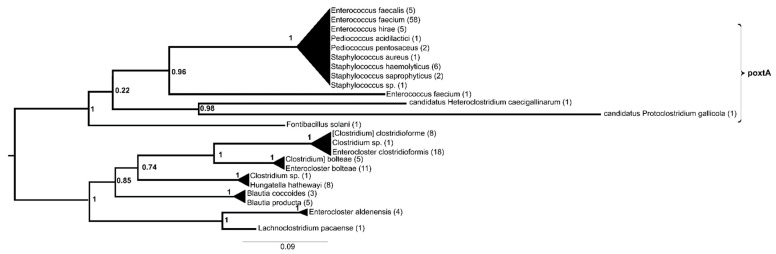
Phylogeny of genes with poxtA (NG_063824.1) as a query reconstructed with FastTree using the amino acid hits of the reference gene sequence with matches of >40% amino acid identity and >85% query coverage. The numbers above represent SH-like aLRT support values.

The protein sequences of poxtA could be divided into two sister groups with very high support values (Figure 3, Supplementary Materials Figure S3). One contained proteins from Lachnospiraceae (*Enterocloster*, *Hungatella* and *Blautia* spp.), while the other consisted of a basally positioned protein from *Fontibacillus solani*, then a protein from *Enterococcus faecium* and a cluster of identical proteins from *E. faecium*, followed by other enterococci (*E. faecalis* and *E. hirae*), *Pediococcus* spp, *Staphylococcus saprophyticus*, *S. haemolyticus* and *S. aureus*. The separation of one protein from *Protoclostridium gallicola* and another from *Heteroclostridium caecigallinarum* was not supported.

## 3. Discussion

Many antibiotics originate from ancient competitive interactions between microorganisms; antibiotic resistance arises in response, either from potential target organisms or from antibiotic producers that develop resistance to protect themselves [20]. Resistant genes evolve as gain-of-function alterations of genes not originally associated with drug resistance and eventually find their way into pathogenic bacteria through horizontal gene transfer, posing an enormous and ever-growing public health problem [20].

This scenario has been proposed for a number of naturally produced antibiotic families [21,22,23,24] but requires additional explanation in the case of synthetic drug families without natural precursors, such as oxazolidinones. In such cases, the selective force may be the use of another group of drugs to which resistance genes are already present and can spread, which, at the same time, also confer cross-resistance to the synthetic drug. Since all mobile genes that confer resistance to linezolid (*cfr* family, *optrA*, *poxtA*) also provide resistance to phenicols [7,15,16], it is conceivable that phenicols were the drugs responsible for their development and spread.

The origin of resistance genes from functional changes is illustrated by the very ancient evolution of beta-lactamases from penicillin-binding proteins or the appearance of *erm*-family 23S rRNA methylases, which have been shown to be an important mechanism of macrolide/lincosamide/Streptogramin B resistance [25,26]. The ancestors of these methylases are housekeeping genes associated with ribosome biogenesis and the regulation of ribosomal functions [27,28]. It has been experimentally demonstrated that these genes are capable of undergoing changes that lead to macrolide resistance [29].

The *cfr* genes, which also methylate 23S rRNA, form a distinct group closely related to the rlmN methylases [30,31], and it is suggested that they evolved from the rlmN enzymes [31], as evidenced by the appearance of rlmN proteins in basal positions in the cfr trees (Figure 1). The group closest to the rlmN proteins/genes and the closest relatives of the *cfr* genes are the S-adenosyl-methionine-dependent methyltransferases of Actinobacteria and some other taxa [31].

The evolutionary path of the cfr proteins could, therefore, be traced back to the rlmN-related methylases of Actinomycetes, possibly derived from a producer of one of the antibiotic groups to which the *cfr* genes confer resistance. Organisms that first acquired the ancestral *cfr* gene probably belonged to Paenibacillaceae or Thermoactinomycetaceae, after which this gene may have passed once to Clostridia, giving rise to *cfr*(C), *cfr*(E) and clostridial cfrs before passing another time to various Bacillales leading to its spread in clinically important species; then *cfr* and the very closely related *cfr*(D) evolved further in Bacillales [32] with the variant *cfr*(B) in *C. difficile* and enterococci and the later variant *clbA* in *Bacillus* spp., which confers a very similar phenotype [32].

The ancestor of *optrA* could be found in either Lachnospiraceae or Erysipelothrichaceae, and environmental Clostridia probably served as vector organisms. Several *optrA* variants have already been detected in *Streptococcus suis* [33]; thus, *S. suis* may have been one of the first pathogenic species to acquire the ancestral gene or gene variants. It is tempting to assume that the cause of the acquisition of *optrA* lies in its association with swine and the antibiotics used in swine, as has been shown in the case of enterococci associated with swine [12,34,35]. Later, *E. faecium* may have acquired one and *E. faecalis* another variant, probably from *S. suis*, while staphylococci and mammaliicocci acquired them later from these clinically relevant enterococci or from *S. suis*. Only one of the sister variants was found in *Campylobacter*.

In contrast, *poxtA* appears to be represented by a single gene variant in clinically relevant bacteria, as all but one of the sequences from human-relevant species were identical. The immediate source of *poxtA* for these clinically relevant bacteria was, similar to the case of *cfr*, probably bacteria associated with the mammalian gut and/or soil, e.g., *Enterocloster*, *Hungatella* or *Blautia*.

Although the use of the reference genome database as the exclusive data source is a limitation, the actual source of cfr methylases could be the antibiotic producers, while bacteria in the soil and animal microbiota could be responsible for its transfer to pathogenic bacteria, similar to the other antibiotic groups. The use of phenicols in livestock can be hypothesized as a common driving force for the appearance and spread of transmissible oxazolidinone resistance, which draws attention to the role of the interplay between the use of antibiotics in veterinary antibiotic use and the development of antibiotic resistance mechanisms relevant to public health, as highlighted in the One Health concept.

## 4. Materials and Methods

### 4.1. Assembly of the Dataset

The amino acid sequences of the genes *cfr* (NG070225.1), *cfr*(B) (KM359438.1), *cfr*(C) (CCL89685.1), *cfr*(D) (CP044318.1:61513-62586), *cfr*(E) (AJ879565.1), *optrA* (MF805732.1:8426-10393) and *poxtA* (NG_063824.1) were selected as representatives of the plasmid-borne oxazolidinone resistance genes. A custom Blast [36] database was compiled containing all available bacterial proteome sequences in the NCBI RefSeq database (https://ftp.ncbi.nlm.nih.gov/genomes/refseq/, accessed on 2 July 2021). Based on the list of available accessions, the fasta files containing the whole proteome sequences were downloaded individually using wget (n = 219,549; accessed on 2 July 2021). The Blast database was formatted using makeblastdb 2.10.1+ after merging the RefSeq proteomes and formatting the fasta definition lines to retain the taxa names and accession numbers. Blastp 2.10.1+ searches were performed with each gene reference sequence as the query and the custom database as the subject. Hits were saved in a tabular format (-outfmt ‘6 qseqid sseqid qstart qend sstart send pident nident length qcovhsp evalue qseq sseq’). The hits were filtered with awk to obtain hits with at least 40% aa identity and at least 85% query coverage.

### 4.2. Phylogenetic Analysis

The sequences obtained were aligned using MAFFT 7.471 [37] with the auto option. The amino acid alignments were used for the phylogenetic reconstruction of gene trees, for which FastTree 2.1.10 [38] was run with default parameters, and statistical robustness was assessed using SH-like local support values. Rooting phylogenetic trees is non-trivial and can be misleading, especially if the wrong outgroup sequence is used. While methods such as rooting with an outgroup, midpoint rooting, and rooting with ancient gene duplications are widely used, the minimal ancestor deviation (MAD) approach has shown promise for accurately identifying different roots in bacterial phylogenies without outgroup sequences [39]. In addition, MAD rooting has been used to account for uncertainties in gene tree rooting, further highlighting its robustness and accuracy in phylogenetic analyses [40]. Therefore, MAD 2.2 [41] was used to root the phylogenetic trees, which were then visualized using FigTree 1.4.4 (available at http://tree.bio.ed.ac.uk/software/figtree/) before they were further edited in Inkscape 0.92.4 to improve readability.

## Data Availability

The GenBank accession numbers used in this study are available in the Supplementary Materials. Further details are available on request from the corresponding authors.

## Supplementary Materials

The following supporting information can be downloaded at: https://www.mdpi.com/article/10.3390/antibiotics13040311/s1, Figure S1: Extended tree of cfr; Figure S2: Extended tree of optrA; Figure S3: Extended tree of poxtA.
