# Supplementary figures and images for: Phylogeny of Transferable Oxazolidinone Resistance Genes and Homologs
Source: Antibiotics (Basel). 2024 Mar 28;13(4):311. doi: 10.3390/antibiotics13040311 (PMC11047308; doi:10.3390/antibiotics13040311)

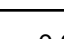

Supplement: Supplementary file 1 [file antibiotics-13-00311-s001.zip › Figure S1 Extended tree of cfr.pdf]

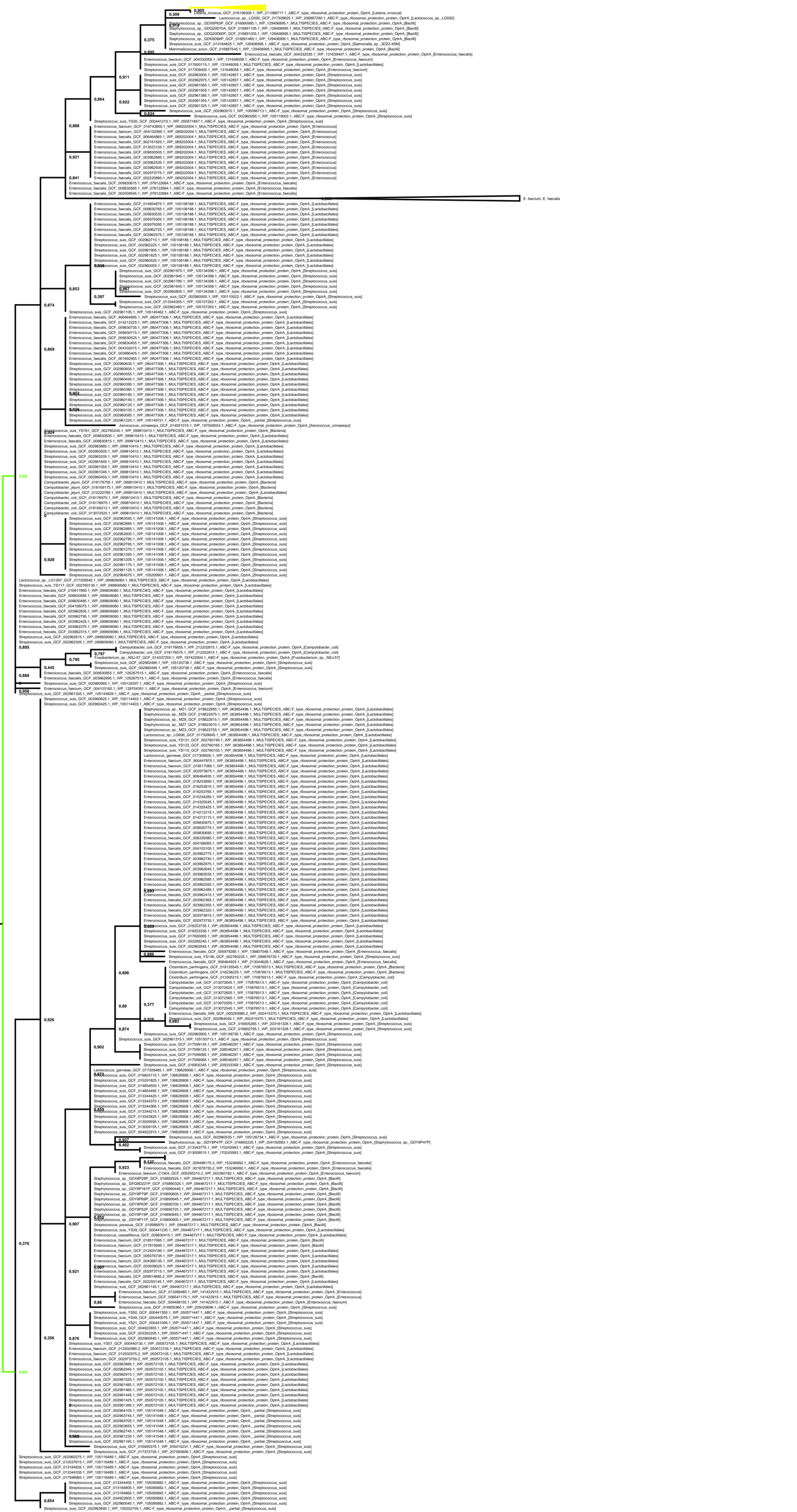

Supplement: Supplementary file 1 [file antibiotics-13-00311-s001.zip › Figure S2 Extended tree of optrA.pdf]

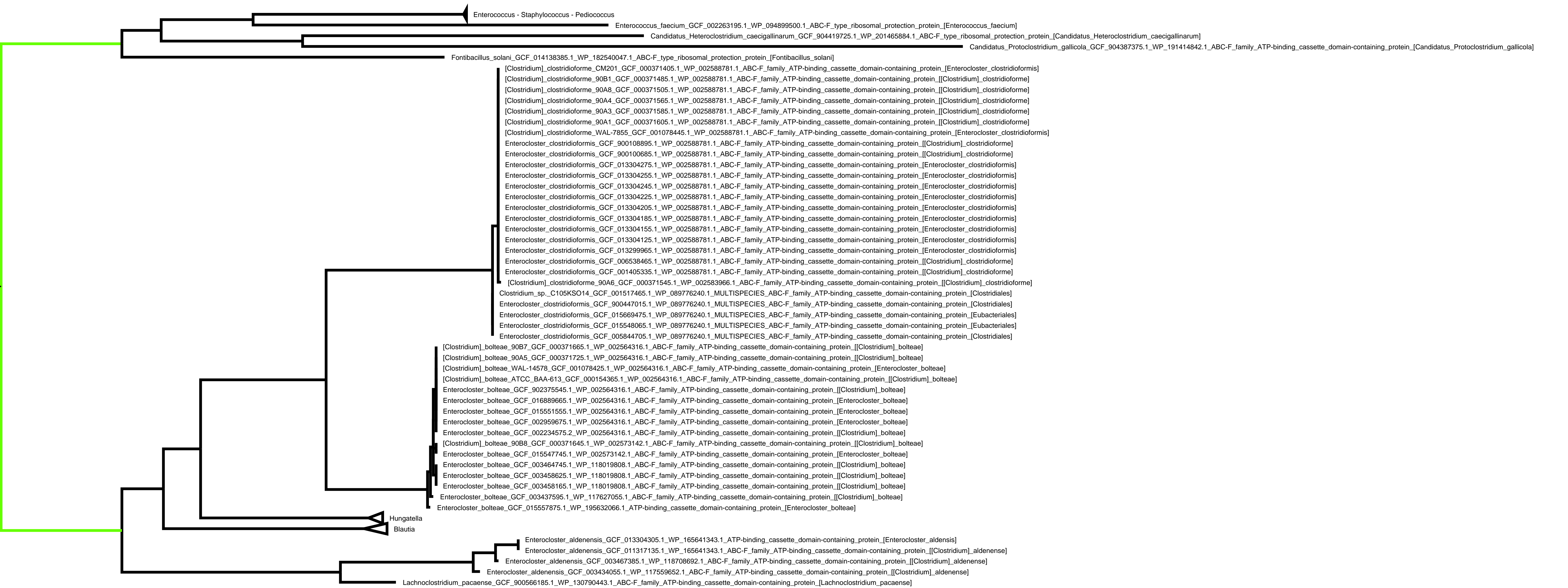

0.09

Supplement: Supplementary file 1 [file antibiotics-13-00311-s001.zip › Figure S3 Extended tree of poxtA.pdf]
